# Supplementary material for: Evolutionary conservation of within-family biodiversity patterns
Source: Nat Commun. 2020 Feb 14;11:882. doi: 10.1038/s41467-020-14720-3 (PMC7021778; doi:10.1038/s41467-020-14720-3)
Supplement: Supplementary file 1 — Supplementary Information [file 41467_2020_14720_MOESM1_ESM.pdf]

Supplementary Information

Evolutionary conservation of within-family biodiversity patterns

Laiolo et al.

Table of Contents

Item	Description
Supplementary Fig. 1.	Biogeographic and evolutionary background of the study
Supplementary Fig. 2.	Likelihood ratio statistics density distributions for diversity variables
Supplementary Fig. 3.	Shapes of the α-species richness trends along elevation for the study families
Supplementary Fig. 4.	Relationships between Edf-α of confamilial communities and the incidence of interactions with autotrophs
Supplementary Table 1.	Phylogenetic signal in diversity variables, organismal and higher level features
Supplementary Note 1.	Study datasets
Supplementary Table 2.	Divergence times between families
Supplementary Note 2.	Organismal level traits
Supplementary Table 3.	Relationship between β-diversity and spatial, elevation and habitat differences in the study families
Supplementary Table 4.	Complete statistical results of the best generalized least square regression models explaining variation in phylogenetically-conserved diversity variables in response to organismal features.

Supplementary Table 5.	List of generalized least square regression models testing for the influence of organismal features on Edf-α in which raw estimates were substituted with 0 values when elevational trends were not fully significant
Supplementary References.	Literature cited in Fig. 1, Note 1, 2, and Table 2

Supplementary Fig. 1. Biogeographic and evolutionary background of the study. **A** Study area and sampling plots. The survey plots layer and the digital elevation layer of the Iberian Peninsula[1] were created with QGIS 2.18.3[2] . QGIS is licensed under Creative Commons Attribution-ShareAlike 3.0 licence (CC BY-SA) https://creativecommons.org/licenses/by-sa/3.0/, no changes were made. **B** Time-calibrated phylogenetic tree of families (Ma = million years) obtained from divergence times (Supplementary Table 2). For representative purposes only, family of the three major taxa are represented in the same phylogenetic tree.

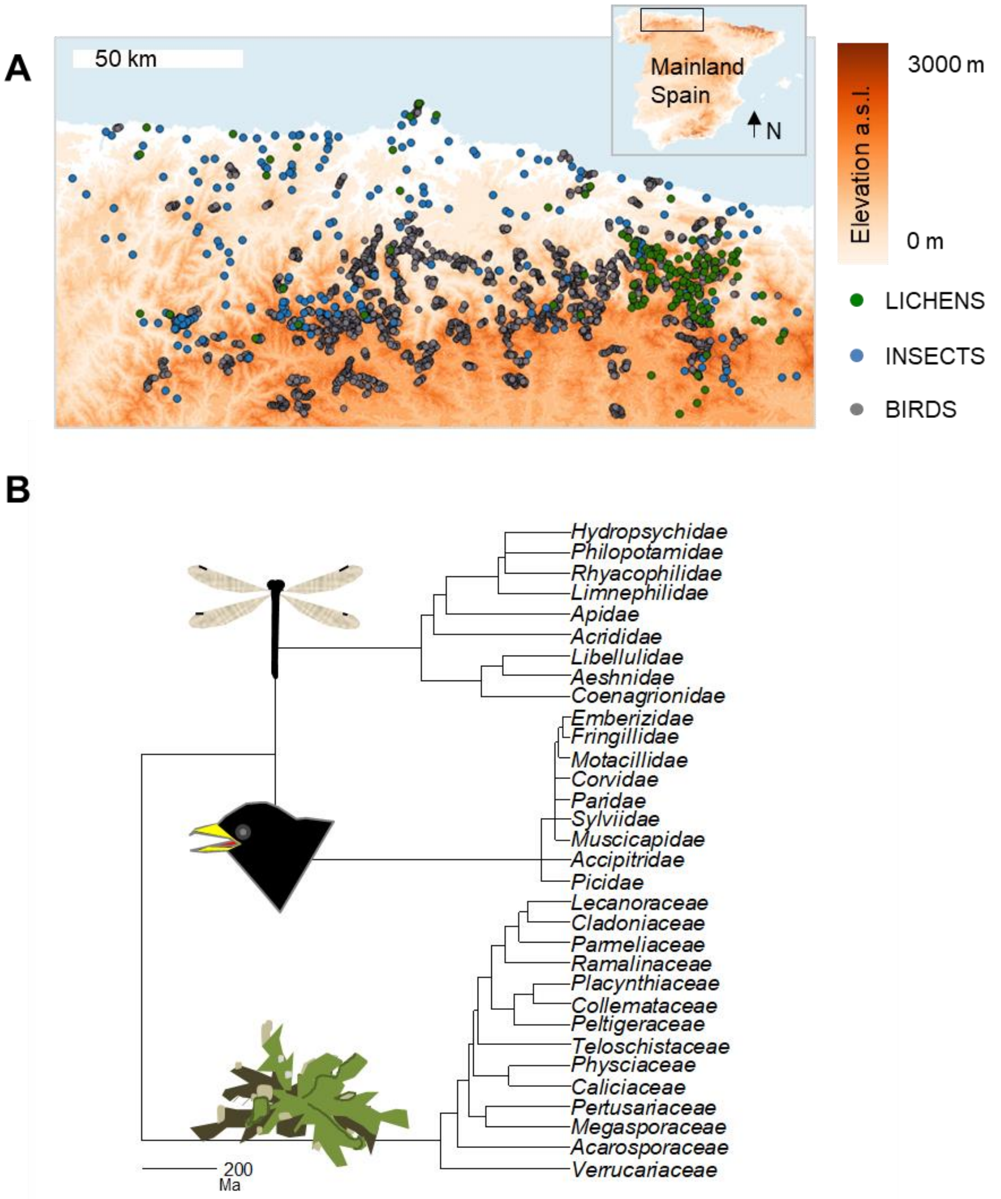

Supplementary Fig. 2. Likelihood ratio statistics density distributions. Density plots depicting the distribution of the likelihood ratio statistics δ for two model comparison, a null model with random draws independent of the phylogeny (white noise WN model, lighter density distributions) and a Brownian motion BM model of evolution (darker density distributions). The dashed vertical line indicates the observed value of δ when the models are fit to our dataset. A total of 1000 replicates were used for each distribution. Acronyms as in Figure 1.

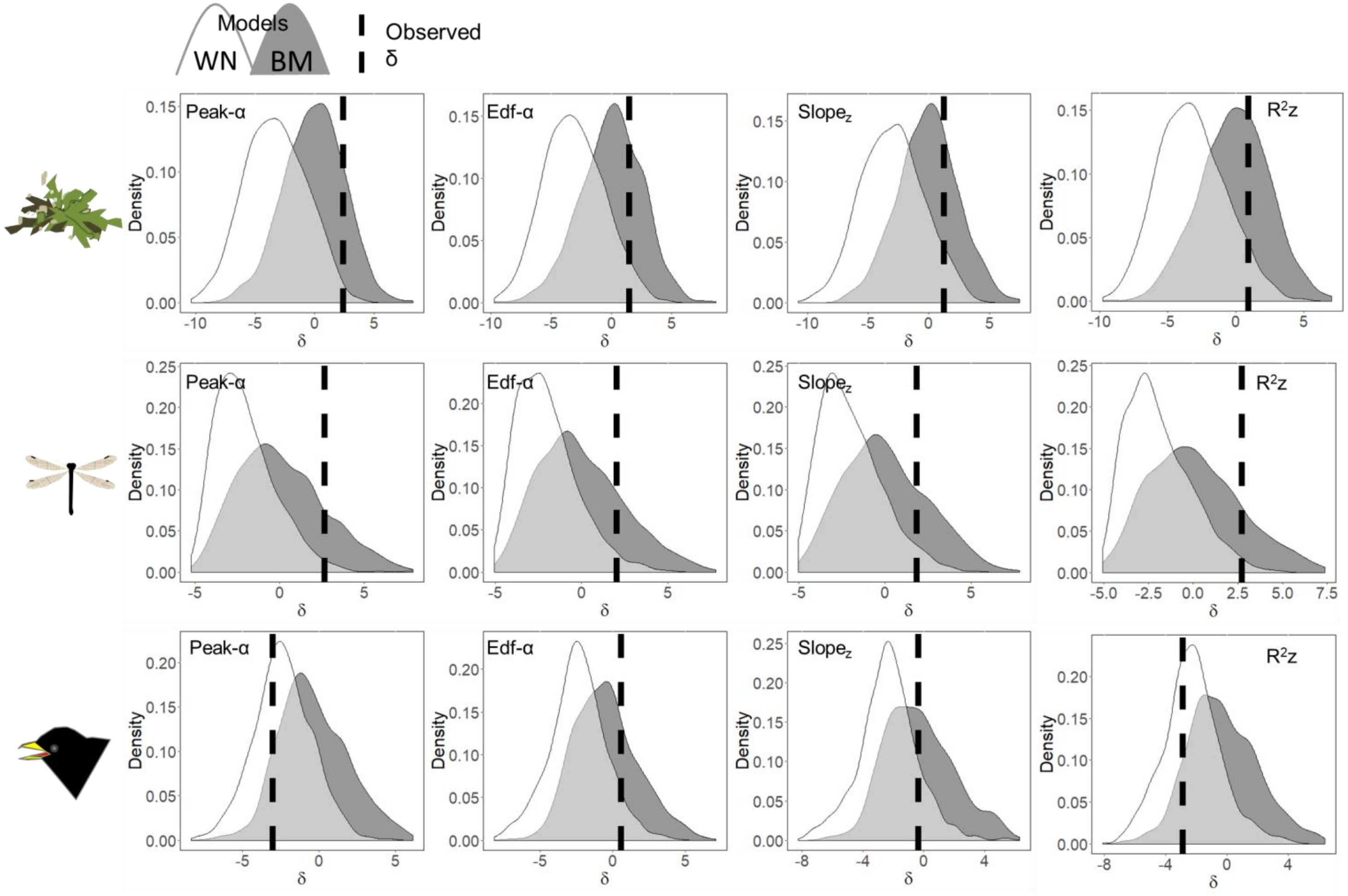

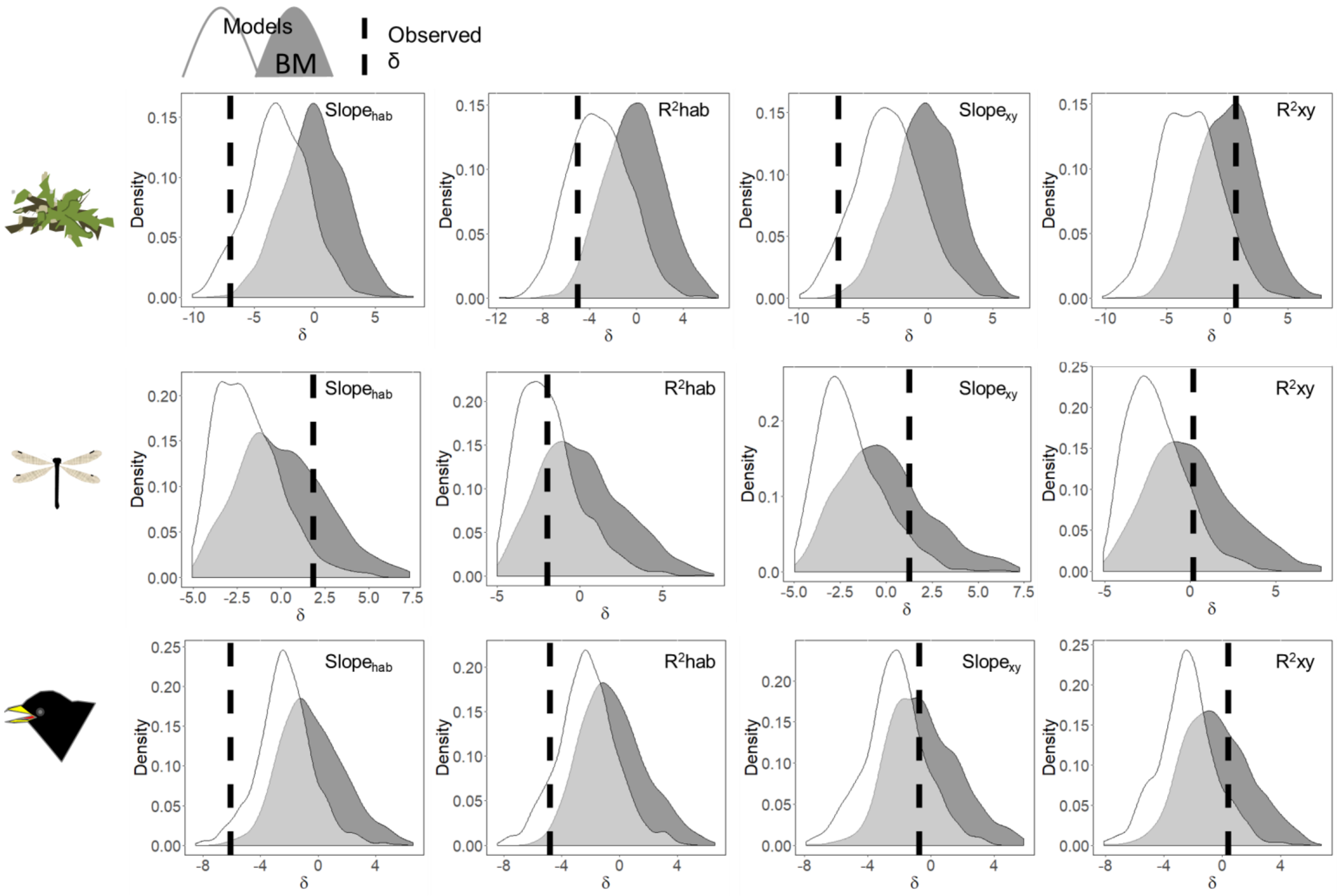
Models
BM
Observed δ
Slope_hab
R²hab
Slope_xy
R²xy
Density
δ

Supplementary Fig. 3. Shapes of the α-species richness trends along elevation for the study families. Curves were obtained by means of generalised additive models fitting Log(species richness +1) on elevation, controlling for geographic coordinates in those cases in which families displayed significant latitudinal or longitudinal trends (in parentheses in the x-axis). Dotted lines represent 95% CI: Non-significant trends (n.s. in plots) were found in some families. Below families' names, the elevation at which species richness peaks and CI were reported (estimated by piecewise regressions, with inflection points bounded in intervals of ± 500 m around the peak in plots). Large CI and standard errors resulted for diversity responses with poorly defined peaks; including these errors in analyses of phylogenetic signal permitted to weight data for uncertainty in diversity-elevation patterns.

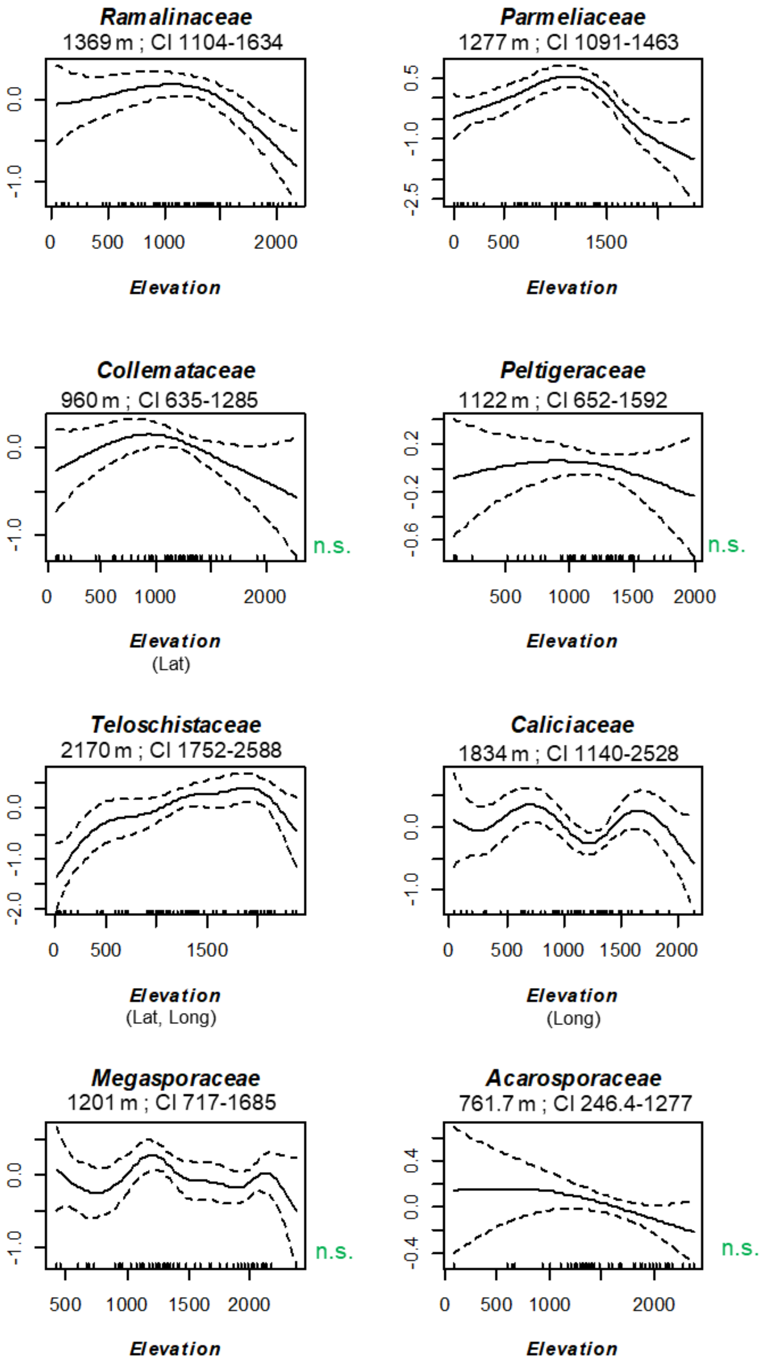

Ramalinaceae
1369 m ; CI 1104-1634
Elevation
Parmeliaceae
1277 m ; CI 1091-1463
Elevation
Cladoniaceae
1364 m ; CI 991-1737
Elevation
Lecanoraceae
1405 m ; CI 1096-1714
Elevation
Collemataceae
960 m ; CI 635-1285
n.s.
Elevation
(Lat)
Peltigeraceae
1122 m ; CI 652-1592
n.s.
Elevation
Placynthiaceae
960.4 m ; CI 291.8-1629
n.s.
Elevation
(Long)
Physciaceae
1347 m ; CI 1178-1516
Elevation
Teloschistaceae
2170 m ; CI 1752-2588
Elevation
(Lat, Long)
Caliciaceae
1834 m ; CI 1140-2528
Elevation
(Long)
Verrucariaceae
2043 m ; CI 1766-2320
Elevation
(Lat)
Pertusariaceae
1119 m ; CI 940-1298
Elevation
(Long)
Megasporaceae
1201 m ; CI 717-1685
n.s.
Elevation
Acarosporaceae
761.7 m ; CI 246.4-1277
n.s.
Elevation

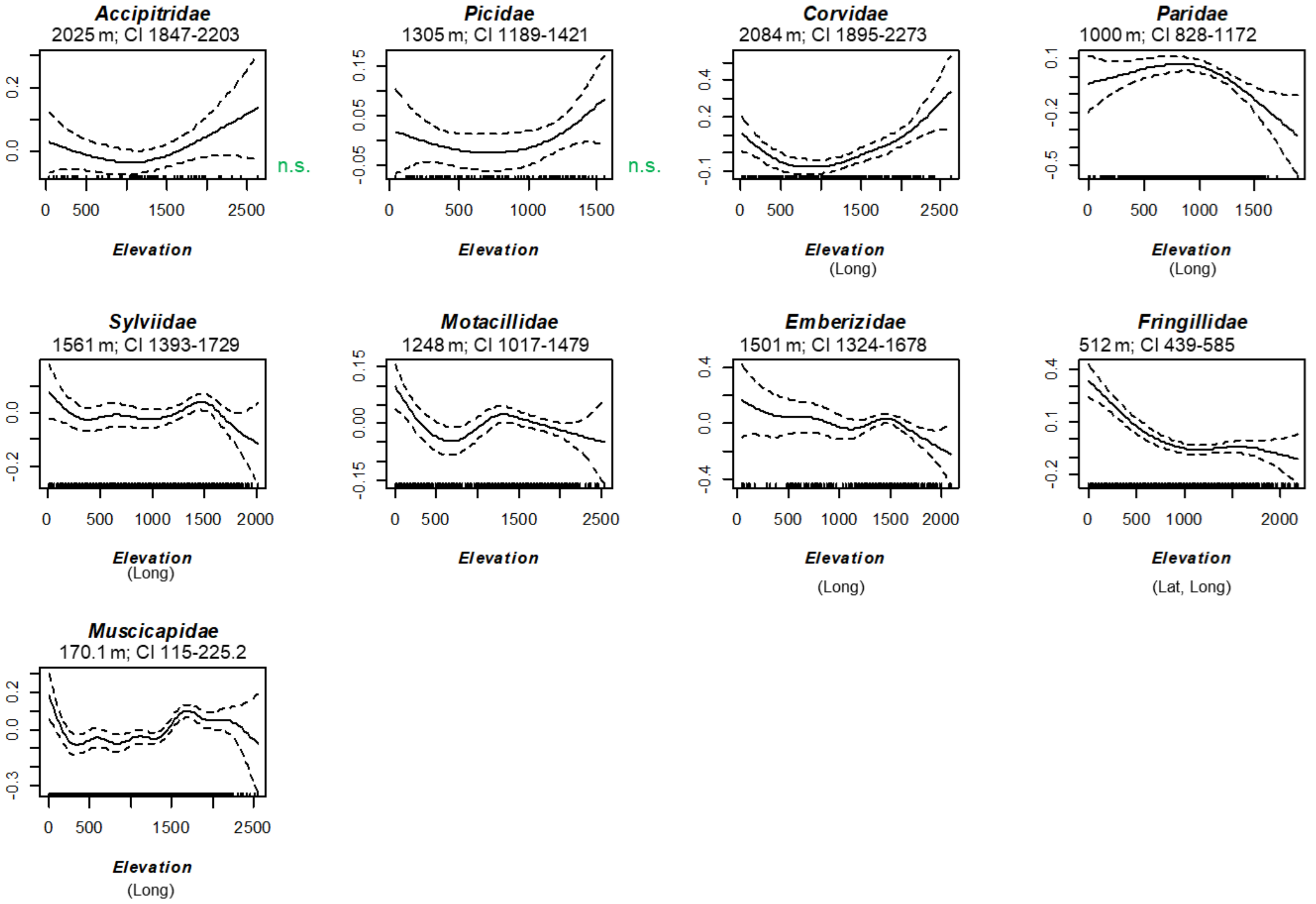
Accipitridae
2025 m; CI 1847-2203
n.s.
Elevation
Picidae
1305 m; CI 1189-1421
n.s.
Elevation
Corvidae
2084 m; CI 1895-2273
Elevation
(Long)
Paridae
1000 m; CI 828-1172
Elevation
(Long)
Sylviidae
1561 m; CI 1393-1729
Elevation
(Long)
Motacillidae
1248 m; CI 1017-1479
Elevation
Emberizidae
1501 m; CI 1324-1678
Elevation
(Long)
Fringillidae
512 m; CI 439-585
Elevation
(Lat, Long)
Muscicapidae
170.1 m; CI 115-225.2
Elevation
(Long)

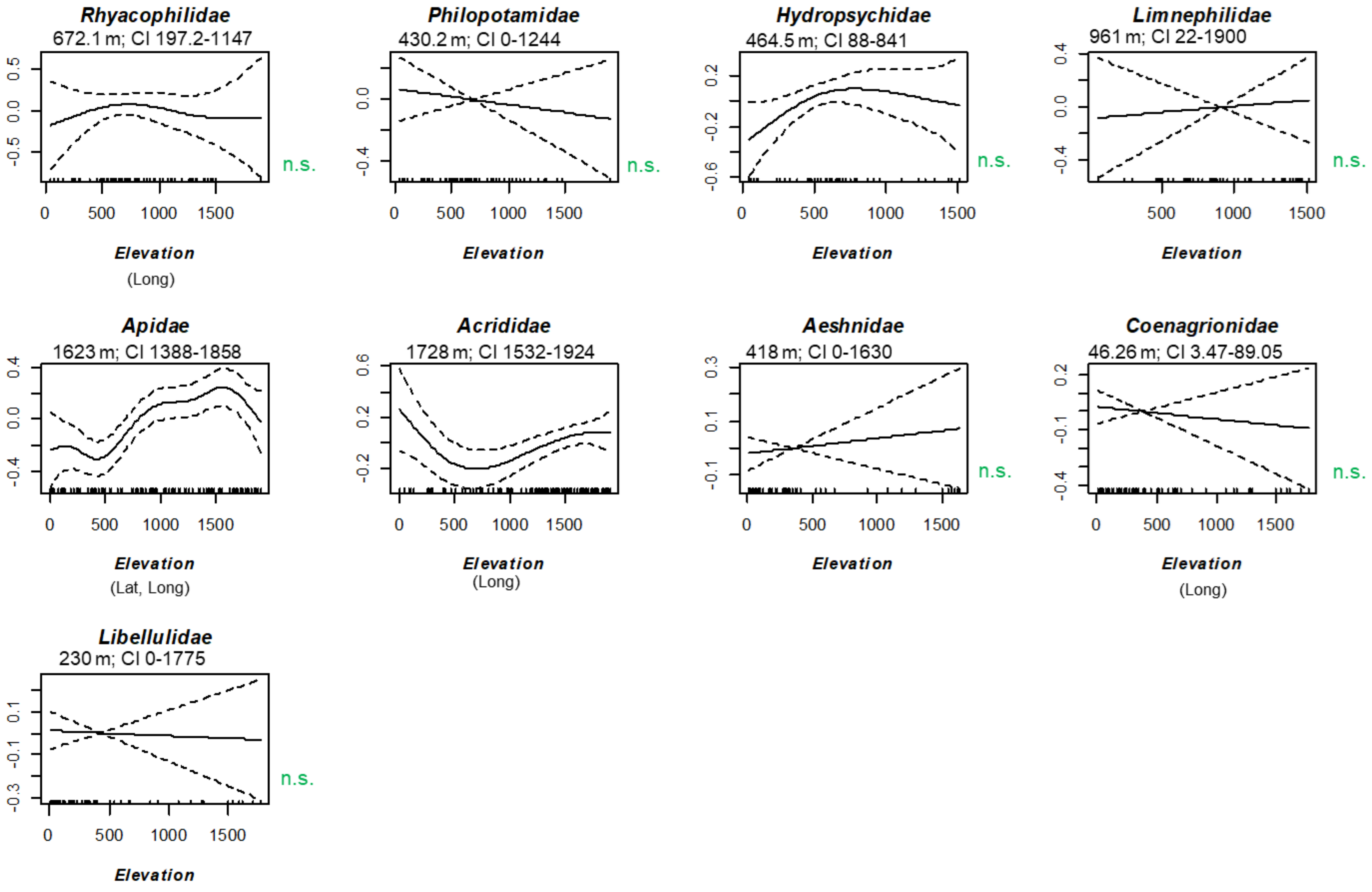
Rhyacophilidae
672.1 m; CI 197.2-1147
n.s.
Elevation
(Long)
Philopotamidae
430.2 m; CI 0-1244
n.s.
Elevation
Hydropsychidae
464.5 m; CI 88-841
n.s.
Elevation
Limnephilidae
961 m; CI 22-1900
n.s.
Elevation
Apidae
1623 m; CI 1388-1858
Elevation
(Lat, Long)
Acrididae
1728 m; CI 1532-1924
Elevation
(Long)
Aeshnidae
418 m; CI 0-1630
n.s.
Elevation
Coenagrionidae
46.26 m; CI 3.47-89.05
n.s.
Elevation
(Long)
Libellulidae
230 m; CI 0-1775
n.s.
Elevation

Supplementary Fig. 4. Relationships between Edf-α of confamilial communities and the incidence of interactions with autotrophs. Scatterplots of the relationships between the parameters of the smoothing function Edf-α of confamilial communities, in the y-axis, and measures of the incidences of green algal endosymbiosis in lichens and of trophic interactions with plants in animals, on the x-axis. In the upper row, we present relationships with Edfs estimated irrespective of the significance of the elevation trends, in the lower row, relationships for Edfs in which raw values were substituted with 0 values when generalized additive models were not fully significant (i.e. no estimated parameters; Edf-α (0 corr.) in graphs). Rs = Spearman correlation coefficient. Each point represents a family.

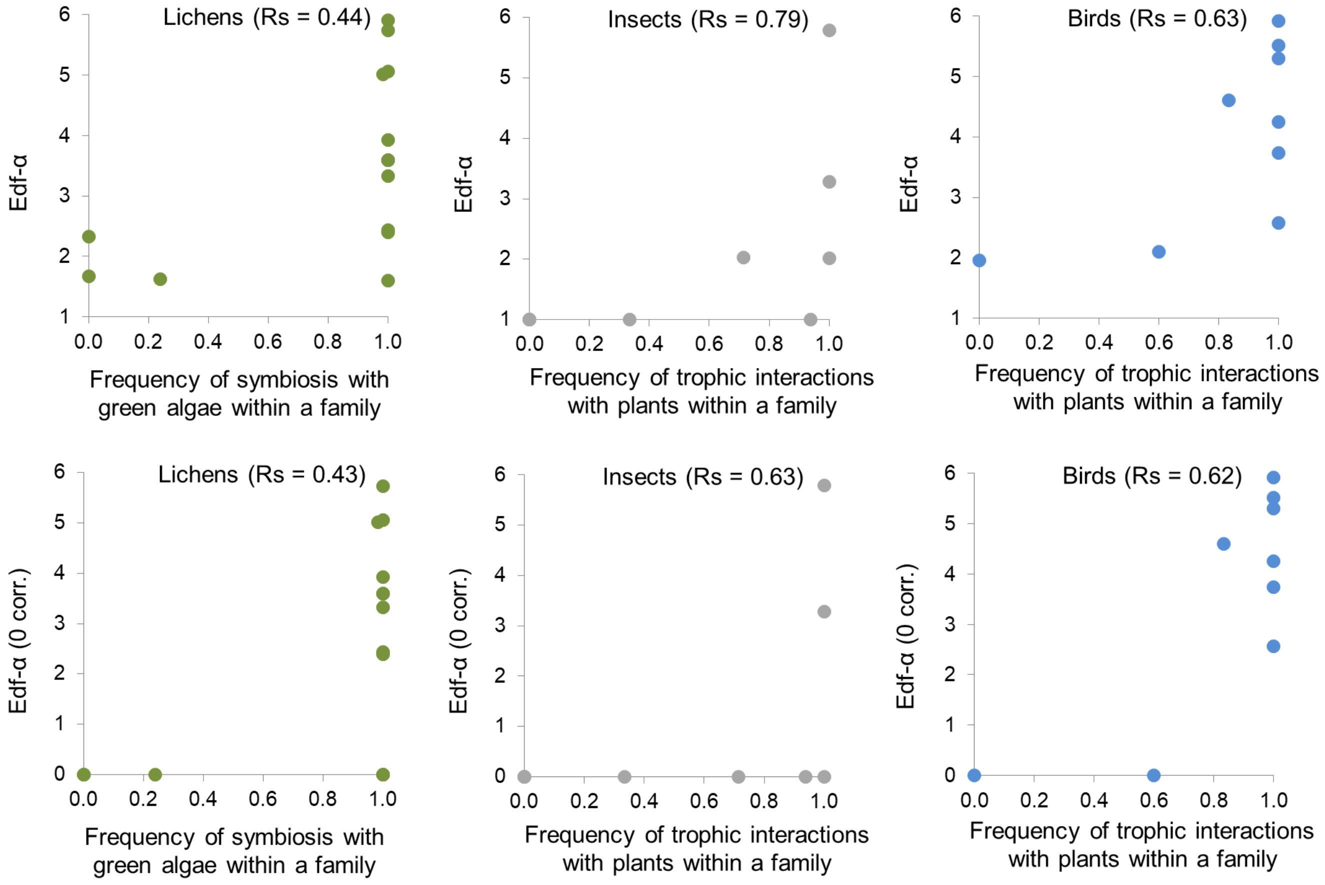

Supplementary Table 1. Phylogenetic signal in diversity variables, organismal and higher level features. Signals were estimated by means of Blomberg's statistics and regressions on distance matrices, with 1000 simulations/permutations. Blomberg K, the coefficient of determination of regressions R^2, and F and P values (in parenthesis) are shown. Significant signals are depicted in bold. All significant coefficients of regression on distance matrices have a positive sign: dissimilarity between clades increases with node age. The variable Edf-α 0 corr. indicate the corrected values of the effective number of parameters of the smoothing function (Edfs were set to 0 were when generalized additive models were not fully significant). Sample size corresponds to the number of families.

	Lichens (14 families)		Insects (9 families)		Birds (9 families)	
**Diversity variables**	Blomberg statistic K (P)	Regression on distance matrices pseudo-R^2 (F, P)	Blomberg statistic K (P)	Regression on distance matrices pseudo-R^2 (F, P)	Blomberg statistic K (P)	Regression on distance matrices pseudo-R^2 (F, P)
Elevation at which species richness peaks (Peak-α)	**1.16 (0.046)**	**0.28 (35.2; 0.006)**	**0.92 (0.045)**	**0.09 (3.5: 0.049)**	0.66 (0.62)	0.005 (0.18; 0.74)
Complexity of the elevational richness profile (Edf-α)	**1.05 (0.044)**	**0.06 (5.6; 0.045)**	**1.21 (0.029)**	**0.23 (4.9; 0.03)**[1]	**1.20 (0.022)**	**0.19 (8.0; 0.013)**
Complexity of the elevational richness profile (Edf-α 0 corr.)	**1.15 (0.022)**	**0.05 (4.6; 0.026)**	**1.34 (0.025)**	**0.09 (3.4; 0.003)**	**1.89 (0.023)**	**0.57(46.8; 0.0007)**
Steepness of spatial turnover ($Slope_{xy}$)	1.39 (0.71)	0.11 (11.7; 0.068)	1.00 (0.062)	0.09 (3.2; 0.07)	0.82 (0.39)	0.006 (0.2; 0.79)
Magnitude of spatial turnover (R^2_{xy})	1.00 (0.08)	0.01 (1.1; 0.37)	0.94 (0.14)	0.03 (1.1; 0.35)	0.96 (0.11)	0.14 (5.9; 0.07)
Steepness of elevational turnover ($Slope_z$)	**1.28 (0.049)**	0.001 (0.1; 0.83)	1.06 (0.16)	0.19 (3.8; 0.06)[1]	**2.00 (0.017)**	**0.41 (24; 0.038)**
Magnitude of elevational turnover (R^2_z)	**1.13 (0.039)**	0.04 (3.6; 0.17)	**1.38 (0.016)**	**0.12 (4.5; 0.031)**	0.70 (0.36)	0.01 (0.4; 0.86)
Steepness of habitat turnover ($Slope_{hab}$)	0.82 (0.75)	0.0005 (0.1; 0.92)	**1.34 (0.049)**	**0.19 (7.9; 0.007)**[1]	0.63 (0.49)	0.04 (1.6; 0.37)
Magnitude of habitat turnover (R^2_{hab})	0.63 (0.84)	0.01 (3.8; 0.38)	0.76 (0.40)	0.04 (1.5; 0.23)	0.57 (0.83)	0.01 (0.4; 0.69)
**Organismal or higher level features**						
Regional species richness	0.83 (0.30)	0.00 (0.00; 1.00)	1.04 (0.08)	0.03 (1.1; 0.25)	0.70 (0.49)	0.01 (0.4; 0.85)
Distribution (n. occupied plots)	0.68 (0.65)	0.001 (0.1; 0.84)	1.09 (0.07)	0.04 (1.5; 0.44)	1.03 (0.11)	0.11 (3.9; 0.17)

Co-occurrence patterns between families	-	0.002 (0.2; 0.78)	-	0.10 (2.3; 0.16)	-	0.13 (5.2; 0.23)
Body size	-	-	**1.96 (0.001)**	**0.30 (14.8; 0.002)**	**1.37 (0.044)**	**0.12 (4.8; 0.043)**
Size-corrected wing length	-	-	**1.72 (0.002)**	0.09 (3.4; 0.07)	0.20 (0.41)	0.07 (2.4; 0.28)
Trophic interactions with plants	-	-	**1.31 (0.036)**	**0.27 (12.2; 0.020)**	**2.05 (0.033)**	**0.65 (66; 0.023)**
Trophic interactions with invertebrates	-	-	0.89 (0.205)	0.02 (0.6; 0.43)	1.10 (0.76)	0.37 (20; 0.24)
Trophic interactions with vertebrates	-	-	-	-	0.89 (0.096)	0.06 (2.0; 0.25)
Endolithic growth form	**1.69 (0.019)**	**0.44 (70; 0.002)**	-	-	-	-
Crustose growth form	0.63 (0.81)	0.02 (2.0; 0.18)	-	-	-	-
Squamulose growth form	0.56 (0.90)	0.01 (0.1; 0.66)	-	-	-	-
Foliose growth form	0.68 (0.67)	0.05 (4.6; 0.19)	-	-	-	-
Fruticose growth form	0.66 (0.68)	0.05 (4.7; 0.25)	-	-	-	-
Mainly sexual reproduction	0.78 (0.39)	0.000 (0.04; 0.87)	-	-	-	-
Mainly asexual by soredia or soredia-like structures	0.85 (0.39)	0.009 (0.9; 0.54)	-	-	-	-
Mainly asexual by isidia or isidia-like structures	1.22 (0.17)	0.02 (1.8; 0.48)	-	-	-	-
Mainly asexual by thallus fragmentation	1.20 (0.68)	0.001 (0.04; 0.85)	-	-	-	-
Symbiosis with green alga	**1.52 (0.018)**	0.006 (0.6; 0.75)	-	-	-	-
Symbiosis with cyanobacteria	**1.51 (0.019)**	0.004 (0.3; 0.77)	-	-	-	-

[1] Quadratic relationship

Supplementary Note 1: Study datasets

Field survey and literature data

We compiled a database for 122 localities in the Cantabrian Mountains where lichens were surveyed, in the elevational range of 0-2400 m a.s.l., from Lopez de Silanes Vázquez et al.[3,4], De La Torre Fernández & Fernández Ordóñez [5] and Aragón et al.[6]. We selected data on geographically defined localities in which all lichen species were systematically recorded, and excluded checklists of broad areas and punctual sampling when referring to a few lichen groups.

We personally sampled breeding birds in 2347 plots from sea level to 2600 m a.s.l., by means of point counts in 100-m-radius circular plots as detailed in Laiolo et al.[7,8]. We built an insect dataset with data from four orders: Odonata[9], Trichoptera[10-16], Orthoptera of the family Acrididae (personal sampling), and Hymenoptera of the family Apidae (personal sampling). Overall, this dataset included 530 plots from sea level to 1900 m a.s.l., and again we excluded, from published surveys, punctual sampling of a few groups and checklists of broad areas. When surveys were repeated across seasons and years, as the case of Odonata, we selected a sole sampling date for data to be comparable with the rest of insects. Details on sampling methods for Orthoptera and Hymenoptera can be found in Laiolo et al. [8,17]. In the Iberian Peninsula, Hymenoptera Apidae are represented by bumblebees (Bombinae) and the honeybee *Apis mellifera*[18]. The honeybee is managed and its occurrence depends on human activity and anthropogenic hive location[19]; thus, we excluded it from sampling for the sake of studying the composition of natural communities.

Geographic coordinates, elevation and cover of different habitats types (water bodies, grass, shrubs, rocks and trees) in a radius of 100 m around the plot centre were obtained in the field for birds. We estimated the percent cover of trees, shrubs, grass, rocks and water bodies from aerial photographs (www.ign.es/ign_iberpix/) for lichens and insects. All data are available in the institutional repository of the Spanish National Research Council DIGITAL CSIC, DOI: http://dx.doi.org/10.20350/digitalCSIC/10529.

Sampling biases

As previously mentioned, the entire lichen or bird community was surveyed in the respective sites for these taxa. Therefore, the families of these groups experienced the same gradient of environmental conditions, and we searched for all families in each locality. In the case of the mega-diverse insect class, plots included partial surveys of one of the four groups: (1) Apidae family, (2) Acrididae family, (3) Odonata families, and (4) Trichoptera families. The use of four independent datasets can gave rise to biases, given that differences among sampling localities (e.g., range of elevation, latitude or longitude) could potentially influence diversity responses of these taxa. For instance, families sampled in a wider range of elevations may display sharper elevational turnover than families sampled in a more limited range, and similarities among families might arise due to this bias. To control for this, we analysed the phylogenetic signal in three variables potentially biased by sampling: elevation range, latitudinal range and longitudinal range. If these variables are affected by phylogeny, then the significant phylogenetic signal of diversity responses (the main objective of this study) may be an artefact of the phylogenetic structure of the sampling protocol. Among these variables, we found that only two had significant phylogenetic signal: the latitudinal range ($K = 1.95$, $P = 0.004$) and the longitudinal range ($K = 1.14$, $P = 0.032$). The former was largest and the latter smaller in the Odonata, a taxon that is also the earliest diverging group (Supplementary Fig. 1). To assess whether this influenced the phylogenetic structure of diversity responses, we performed generalised least square analyses with phylogenetically conserved diversity responses as dependent variables, and these two variables and phylogenetic structure as predictor variables (See Methods). We found that models including phylogeny performed better than models excluding it, thus we could reasonably assume that phylogeny affects diversity responses when controlling for biases in sampling[20]. Moreover, when significant latitudinal and longitudinal patterns in species richness were found, we controlled for variation across latitude and longitude to estimate diversity variables, thus variation in these geographic features, if any, was minimized when controlling for spatial autocorrelation.

Supplementary Table 2. Divergence times. Matrix of pairwise patristic distances among families. Distances were obtained from the TimeTree database[21] and are expressed in millions of years. Information downloaded from http://www.timetree.org/; January 2019.

	Megasporaceae	Cladoniaceae	Lecanoraceae	Parmeliaceae	Ramalinaceae	Teloschistaceae	Verrucariaceae	Collemataceae	Peltigeraceae	Pertusariaceae	Caliciaceae	Physciaceae	Acarosporaceae	Placynthiaceae	Fringillidae	Motacillidae	Paridae	Sylviidae	Corvidae	Picidae	Accipitridae	Emberizidae	Muscicapidae	Rhyacophilidae	Philopotamidae	Hydropsychidae	Limnephilidae	Apidae	Acrididae	Coenagrionidae	Aeshnidae	Libellulidae
Megasporaceae	0	544	544	544	544	544	700	544	544	458	544	544	610	544	2300	2300	2300	2300	2300	2300	2300	2300	2300	2300	2300	2300	2300	2300	2300	2300	2300	2300
Cladoniaceae	544	0	232	276	358	500	700	430	430	544	518	518	610	430	2300	2300	2300	2300	2300	2300	2300	2300	2300	2300	2300	2300	2300	2300	2300	2300	2300	2300
Lecanoraceae	544	232	0	**276**	358	500	700	430	430	544	518	518	610	430	2300	2300	2300	2300	2300	2300	2300	2300	2300	2300	2300	2300	2300	2300	2300	2300	2300	2300
Parmeliaceae	544	**276**	**276**	0	358	500	700	430	430	544	518	518	610	430	2300	2300	2300	2300	2300	2300	2300	2300	2300	2300	2300	2300	2300	2300	2300	2300	2300	2300
Ramalinaceae	544	358	358	358	0	500	700	430	430	544	518	518	610	430	2300	2300	2300	2300	2300	2300	2300	2300	2300	2300	2300	2300	2300	2300	2300	2300	2300	2300
Teloschistaceae	544	500	500	500	500	0	700	500	500	544	518	518	610	500	2300	2300	2300	2300	2300	2300	2300	2300	2300	2300	2300	2300	2300	2300	2300	2300	2300	2300
Verrucariaceae	700	700	700	700	700	700	0	700	700	700	700	700	700	700	2300	2300	2300	2300	2300	2300	2300	2300	2300	2300	2300	2300	2300	2300	2300	2300	2300	2300
Collemataceae	544	430	430	430	430	500	700	0	302	544	518	544	610	203	2300	2300	2300	2300	2300	2300	2300	2300	2300	2300	2300	2300	2300	2300	2300	2300	2300	2300
Peltigeraceae	544	430	430	430	430	500	700	302	0	544	518	518	610	302	2300	2300	2300	2300	2300	2300	2300	2300	2300	2300	2300	2300	2300	2300	2300	2300	2300	2300
Pertusariaceae	458	544	544	544	544	544	700	544	544	0	544	544	610	544	2300	2300	2300	2300	2300	2300	2300	2300	2300	2300	2300	2300	2300	2300	2300	2300	2300	2300
Caliciaceae	544	518	518	518	518	518	700	518	518	544	0	332.8	610	518	2300	2300	2300	2300	2300	2300	2300	2300	2300	2300	2300	2300	2300	2300	2300	2300	2300	2300
Physciaceae	544	518	518	518	518	518	700	544	518	544	332	0	610	544	2300	2300	2300	2300	2300	2300	2300	2300	2300	2300	2300	2300	2300	2300	2300	2300	2300	2300
Acarosporaceae	610	610	610	610	610	610	700	610	610	610	610	610	0	610	2300	2300	2300	2300	2300	2300	2300	2300	2300	2300	2300	2300	2300	2300	2300	2300	2300	2300
Placynthiaceae	544	430	430	430	430	500	700	203	302	544	518	544	610	0	2300	2300	2300	2300	2300	2300	2300	2300	2300	2300	2300	2300	2300	2300	2300	2300	2300	2300
Fringillidae	2300	2300	2300	2300	2300	2300	2300	2300	2300	2300	2300	2300	2300	2300	0	66	84	84	82	162	160	41.8	84	1588	1588	1588	1588	1588	1588	1588	1588	1588
Motacillidae	2300	2300	2300	2300	2300	2300	2300	2300	2300	2300	2300	2300	2300	2300	66	0	84	84	82	162	160	66	84	1588	1588	1588	1588	1588	1588	1588	1588	1588
Paridae	2300	2300	2300	2300	2300	2300	2300	2300	2300	2300	2300	2300	2300	2300	84	84	0	85.4	82	162	160	84	85.4	1588	1588	1588	1588	1588	1588	1588	1588	1588
Sylviidae	2300	2300	2300	2300	2300	2300	2300	2300	2300	2300	2300	2300	2300	2300	84	84	85.4	0	82	162	160	84	84	1588	1588	1588	1588	1588	1588	1588	1588	1588
Corvidae	2300	2300	2300	2300	2300	2300	2300	2300	2300	2300	2300	2300	2300	2300	82	82	82	82	0	162	160	84	82	1588	1588	1588	1588	1588	1588	1588	1588	1588
Picidae	2300	2300	2300	2300	2300	2300	2300	2300	2300	2300	2300	2300	2300	2300	162	162	162	162	162	0	160	162	162	1588	1588	1588	1588	1588	1588	1588	1588	1588
Accipitridae	2300	2300	2300	2300	2300	2300	2300	2300	2300	2300	2300	2300	2300	2300	160	160	160	160	160	160	0	160	160	1588	1588	1588	1588	1588	1588	1588	1588	1588
Emberizidae	2300	2300	2300	2300	2300	2300	2300	2300	2300	2300	2300	2300	2300	2300	41.8	66	84	84	82	162	160	0	84	1588	1588	1588	1588	1588	1588	1588	1588	1588
Muscicapidae	2300	2300	2300	2300	2300	2300	2300	2300	2300	2300	2300	2300	2300	2300	84	84	85.4	84	82	162	160	84	0	1588	1588	1588	1588	1588	1588	1588	1588	1588
Rhyacophilidae	2300	2300	2300	2300	2300	2300	2300	2300	2300	2300	2300	2300	2300	2300	1588	1588	1588	1588	1588	1588	1588	1588	1588	0	358	358	452	670	738	798	798	798
Philopotamidae	2300	2300	2300	2300	2300	2300	2300	2300	2300	2300	2300	2300	2300	2300	1588	1588	1588	1588	1588	1588	1588	1588	1588	358	0	354	358	670	738	798	798	798
Hydropsychidae	2300	2300	2300	2300	2300	2300	2300	2300	2300	2300	2300	2300	2300	2300	1588	1588	1588	1588	1588	1588	1588	1588	1588	358	354	0	358	670	738	798	798	798
Limnephilidae	2300	2300	2300	2300	2300	2300	2300	2300	2300	2300	2300	2300	2300	2300	1588	1588	1588	1588	1588	1588	1588	1588	1588	452	358	358	0	670	738	798	798	798
Apidae	2300	2300	2300	2300	2300	2300	2300	2300	2300	2300	2300	2300	2300	2300	1588	1588	1588	1588	1588	1588	1588	1588	1588	670	670	670	670	0	738	798	798	798
Acrididae	2300	2300	2300	2300	2300	2300	2300	2300	2300	2300	2300	2300	2300	2300	1588	1588	1588	1588	1588	1588	1588	1588	1588	738	738	738	738	738	0	798	798	798
Coenagrionidae	2300	2300	2300	2300	2300	2300	2300	2300	2300	2300	2300	2300	2300	2300	1588	1588	1588	1588	1588	1588	1588	1588	1588	798	798	798	798	798	798	0	478	478
Aeshnidae	2300	2300	2300	2300	2300	2300	2300	2300	2300	2300	2300	2300	2300	2300	1588	1588	1588	1588	1588	1588	1588	1588	1588	798	798	798	798	798	798	478	0	370
Libellulidae	2300	2300	2300	2300	2300	2300	2300	2300	2300	2300	2300	2300	2300	2300	1588	1588	1588	1588	1588	1588	1588	1588	1588	798	798	798	798	798	798	478	370	0

Supplementary Note 2: Organismal level traits

Animal traits

Body size. We used female weight for birds, and female body length for insects (queens in bees). Bird weight was obtained from the literature (see Laiolo et al.[22]), as well as Odonata body length[9]. In the rest of the insects, measurements were obtained from our collections (bees and grasshoppers) or from the collections of the Departamento de Biología de Organismos y Sistemas of Oviedo University (Trichoptera).

Size-corrected wing length. We measured the ratio between wing length and an indicator of structural body size in each taxa, i.e. tarsus length in birds and total body length in the rest of the taxa (sources as above).

Trophic interactions. Information published by Laiolo et al.[8] was used to obtain information on the trophic items of bird species. For comparative purposes, items were cumulated in three coarse categories: feeding on invertebrates, feeding on vertebrates (including vertebrate carcasses) and on plants (frugivory, herbivory and seed predation). For insects, we also derived broad categories: feeding on plant matter, on invertebrates, vertebrates and detritus. The latter two categories appeared in one, or a few families, with low incidence, and were therefore excluded from analyses. We used personal information for bees and grasshoppers species[8]. For Trichoptera species, we obtained information from Slack[23], Harding[24], Presa et al.[25], Lavandier & Cereghino[26], Cereghino[27], Basaguren et al.[28], Corallini & Bicchierai[29], and the webpage https://trichopteraireland.wordpress.com and literature therein. For Odonates, information was obtained from Galliani et al.[30].

Lichen traits

Growth form. This was classified as crustose (leprose, rimose, aerolate, or placodiomorph), crustose-endolithic, squamulose, foliose (umbilicate or not), fruticose (cup-like, beard-like, shrub-like). Information was derived from *ITALIC 5.0, the Information System on Italian Lichens*[31].

Reproductive strategy and dispersal. This was classified as mainly sexual, mainly asexual by soredia or soredia-like structures, mainly asexual by isidia or isidia-like

structures, or mainly asexual by thallus fragmentation. Information was derived from *ITALIC 5.0, the Information System on Italian Lichens*[31].

Endosymbiont. We obtained information on symbiont genera or higher taxa and then clumped information into three coarse categories: cyanobacteria, green algae and yellow-green algae. The latter, extremely rare, was then omitted in analyses. We completed the information obtained from *ITALIC 5.0, the Information System on Italian Lichens*[31] and the *Consortium of North American Lichen Herbaria* (http://lichenportal.org/cnalh/) with information from Ahmadjian[32], Rambold et al.[33], Beck et al.[34], Helms et al.[35], Meier et al.[36], Dahlman et al.[37], Piercey-Normore[38], Thüs et al.[39], Dal Grande et al.[40], Lindgren et al.[41], Leavitt et al.[42], Sadowsky & Ott[43], Voytsekhovich et al.[44] and Moya et al.[45].

Information was fully available for all avian species, while in the case of lichens and insects it was missing from some species. All data are available in the institutional repository of the Spanish National Research Council DIGITAL CSIC, DOI: http://dx.doi.org/10.20350/digitalCSIC/10529.

Supplementary Table 3. The relationship between β-diversity and spatial, elevation and habitat differences in the study families. Results of regressions of β-diversity matrix on the matrices of geographic distances, elevational and habitat dissimilarities. The column "Coefficients" reports the partial coefficients for each gradient, controlling for turnover in the rest of gradients. The column "Pseudo-R^2" refers to the coefficient of determination of each gradient for separate. The standard errors (error squares) of spatial, elevational and habitat turnover were estimated as coefficient/two-tailed inverse of the Student's t-distribution for the pseudo-Ps and -degrees of freedom of the regression model. Large error squares were associated with non-significant trends. Including these errors in analyses of phylogenetic signal permitted to weight data by uncertainty of slopes values.

Lichens				*Birds*			
**Megasporaceae**	Coefficient	P	Pseudo-R^2	**Fringillidae**	Coefficient	P	Pseudo-R^2
Spatial turnover	0.1582829	0.022	0.04822	Spatial turnover	-0.0048897	0.611	0.00034
Elevational turnover	0.0000916	0.030	0.0294	Elevational turnover	0.0002484	0.001	0.06086
Habitat turnover	0.0638416	0.024	0.02157	Habitat turnover	0.2136385	0.001	0.08665
**Cladoniaceae**				**Motacillidae**			
Spatial turnover	0.1255736	0.003	0.01008	Spatial turnover	-0.0354583	0.001	0
Elevational turnover	0.0000733	0.003	0.03118	Elevational turnover	0.0003691	0.001	0.16935
Habitat turnover	0.0842343	0.001	0.05957	Habitat turnover	0.1057200	0.001	0.03039
**Lecanoraceae**				**Paridae**			
Spatial turnover	0.0667755	0.001	0.04996	Spatial turnover	0.0207116	0.018	0.00093
Elevational turnover	0.0000723	0.001	0.07115	Elevational turnover	0.0001147	0.001	0.01267
Habitat turnover	0.0710393	0.001	0.04838	Habitat turnover	0.0459761	0.001	0.00692
**Parmeliaceae**				**Sylviidae**			
Spatial turnover	-0.0066626	0.721	0.00508	Spatial turnover	0.0392920	0.001	0.00305
Elevational turnover	0.0001229	0.001	0.12425	Elevational turnover	0.0001888	0.001	0.03822
Habitat turnover	0.0279737	0.013	0.03269	Habitat turnover	0.2139289	0.001	0.07971
**Ramalinaceae**				**Corvidae**			
Spatial turnover	0.0081655	0.619	0.00239	Spatial turnover	0.0156805	0.043	0.0012
Elevational turnover	0.0000601	0.001	0.02763	Elevational turnover	0.0001717	0.001	0.06647
Habitat turnover	0.0700932	0.001	0.04542	Habitat turnover	0.1405305	0.001	0.05092
**Teloschistaceae**				**Picidae**			
Spatial turnover	0.1191936	0.001	0.08066	Spatial turnover	0.0099680	0.719	0.00014
Elevational turnover	0.0000927	0.001	0.09513	Elevational turnover	0.0000163	0.496	0.00044
Habitat turnover	0.0670629	0.001	0.05269	Habitat turnover	0.0440957	0.041	0.00274
**Verrucariaceae**				**Accipitridae**			
Spatial turnover	0.1068575	0.001	0.05915	Spatial turnover	0.0587574	0.092	0.00373
Elevational turnover	0.0000905	0.001	0.08305	Elevational turnover	0.0000619	0.035	0.00929
Habitat turnover	0.0595202	0.001	0.04503	Habitat turnover	0.0452208	0.125	0.00475
**Collemataceae**				**Emberizidae**			
Spatial turnover	0.0979273	0.003	0.02484	Spatial turnover	0.0145559	0.228	0.00109
Elevational turnover	0.0000325	0.244	0.00743	Elevational turnover	0.0001371	0.001	0.01135

Habitat turnover	0.0988053	0.001	0.05294
**Peltigeraceae**			
Spatial turnover	0.0216829	0.498	0.00148
Elevational turnover	0.0000378	0.270	0.00354
Habitat turnover	0.0411205	0.017	0.00906
**Pertusariaceae**			
Spatial turnover	0.0920932	0.011	0.05551
Spatial turnover	0.0000521	0.106	0.04116
Habitat turnover	0.0811841	0.001	0.0394
**Caliciaceae**			
Spatial turnover	0.1330971	0.008	0.07235
Elevational turnover	0.0001256	0.020	0.06253
Habitat turnover	0.0736445	0.002	0.01757
**Physciaceae**			
Spatial turnover	0.1146435	0.001	0.05648
Elevational turnover	0.0000711	0.001	0.05428
Habitat turnover	0.0637371	0.001	0.03405
**Acarosporaceae**			
Spatial turnover	0.1488702	0.287	0.008
Elevational turnover	0.0001290	0.001	0.04301
Habitat turnover	0.0144648	0.447	0.0128
**Placynthiaceae**			
Spatial turnover	0.4164664	0.014	0.03032
Elevational turnover	0.0000064	0.904	0.00265
Habitat turnover	0.0304373	0.406	0.00678

Habitat turnover	-0.0350799	0.091	0.00023
**Muscicapidae**			
Spatial turnover	0.0166947	0.005	0.00096
Elevational turnover	0.0001671	0.001	0.04037
Habitat turnover	0.1418036	0.001	0.04034

Insects

**Rhyacophilidae**	Coefficient	P	Pseudo-R^2
Spatial turnover	0.0351145	0.007	0.01039
Elevational turnover	0.0000474	0.010	0.00751
Habitat turnover	0.0009423	0.951	0.00122
**Philopotamidae**			
Spatial turnover	0.0542878	0.268	0.00824
Elevational turnover	0.0000599	0.401	0.01194
Habitat turnover	-0.0203098	0.776	0.00056
**Hydropsychidae**			
Spatial turnover	0.0445995	0.017	0.01604
Elevational turnover	0.0001055	0.006	0.01879
Habitat turnover	0.0054785	0.845	0.00454
**Limnephilidae**			
Spatial turnover	0.0642190	0.001	0.03456
Elevational turnover	0.0000463	0.095	0.00557
Habitat turnover	0.0106298	0.592	0.00248
**Apidae**			
Spatial turnover	0.0290437	0.099	0.02485
Elevational turnover	0.0002169	0.001	0.2624
Habitat turnover	0.0252728	0.091	0.00791
**Acrididae**			
Spatial turnover	0.0269748	0.054	0.01845
Elevational turnover	0.0002207	0.001	0.23205
Habitat turnover	0.0286680	0.073	0.00516
**Coenagrionidae**			
Spatial turnover	0.0518484	0.023	0.00818
Elevational turnover	0.0000643	0.064	0.01031
Habitat turnover	0.1146677	0.041	0.00984
**Aeshnidae**			
Spatial turnover	-0.0287711	0.211	0.00085
Elevational turnover	0.0001499	0.002	0.03413
Habitat turnover	0.1474829	0.007	0.0135
**Libellulidae**			
Spatial turnover	-0.0071628	0.802	0.00003
Elevational turnover	0.0001782	0.001	0.07822
Habitat turnover	-0.0319641	0.641	0.00008

Supplementary Table 4. **List of the best generalized least square regression models explaining variation in phylogenetically-conserved diversity variables in response to organismal features**. Models were ranked on the basis of ΔAICc, and only models separated by < 3 AICc points are shown. The difference in AICc points from the best model, and AICc weight, the probability of a model to be the best one, are shown in columns. In rows, the regression statistics for each predictor is quoted in parenthesis (Estimate; SE; t; P). Models might include or not a phylogenetic structure based on the Brownian motion BM model of evolution. When "BM" appears in the list of predictors, it means that phylogeny is affecting the relationship between organismal features and diversity variables; when "BM" appears alone, the model includes the sole intercept and phylogenetic structure. Peak-α: elevation at which species richness peaks. Edf-α: effective number of parameters of the smoothing function. $Slope_{hab}$: habitat turnover. $Slope_z$: elevational turnover. R^2_z: magnitude of elevational turnover.

Top-ranking models (ΔAICc < 3)	AICc weight	ΔAICc
Lichens: Peak-α		
Endolithic (2912; 1171;2.5; 0.0303) + Algal symbiosis (313; 266;1.17; 0.26); BM	0.84	0.0
Lichens: Edf-α		
Endolithic (-0.57; 6.45; 0.1; 0.93)+ Algal symbiosis (1.96; 1.28; 1.52; 0.15) ; BM	0.29	0.0
Endolithic (2.10; 6.5;0.3; 0.75); BM	0.23	0.6
Algal symbiosis (1.93; 1.18; 1.62; 0.12); BM	0.14	1.5
Endolithic (-0.31; 5.18; 0.06; 0.95) + Algal symbiosis (2.11; 1.03; 2.05; 0.06)	0.12	1.8
Algal symbiosis (2.10; 0.92; 2.25; 0.04)	0.07	2.8
BM	0.07	2.9
Insects: Peak-α		
Size-corrected wing length (-1600; 375; 4.26; 0.005) + Plant Interaction (1816, 128; 14.1; 0.000)	0.48	0.0
Size-corrected wing length (-2099; 575; 3.64; 0.011) + Plant Interaction (1849; 165; 11.2; 0.000); BM	0.37	0.5

Body size (1687; 597; 2.82; 0.03) + Plant Interaction (1842; 188; 976; 0.0001)	0.11	2.9
Insects: Edf-α		
Plant interaction (2.24, 1.14; 196; 0.09); BM	0.24	0.0
Size-corrected wing length (-4.1; 2.12; 1.92; 0.10) + Plant Interaction (2.99; 1.04; 2.86; 0.028); BM	0.18	0.6
BM	0.16	0.7
Size-corrected wing length (2.24; 1.14; 1.96; 0.09); BM	0.08	1.2
Body size (0.07; 2.28; 0.03; 0.97); BM	0.08	2.1
Size-corrected wing length (-3.83; 1.63; 2.34; 0.057) + Plant Interaction (3.83; 0.93; 3.53; 0.12)	0.07	2.4
Plant Interaction (2.17; 1.02; 2.11, 0.07)	0.07	2.5
Insects: R^2_z		
BM	0.84	0.0
Insects: $Slope_{hab}$		
BM	0.76	0.0
Body size (0.11; 0.034; 3.10; 0.017)	0.18	2.9
Birds: Edf-α		
Plant interaction (2.95; 1.25; 2.35; 0.05)	0.24	0.0
BM	0.19	0.4
Plant Interaction (2.47; 2.05; 1.20; 0.26); BM	0.18	0.5
Size-corrected wing length (-1.05; 0.37; 2.79; 0.027); BM	0.14	1.1
Size-corrected wing length (-0.89; 0.46; 1.93; 0.09)	0.10	1.5
Birds: $Slope_z$		
BM	1.00	0.0

Supplementary Table 5. List of generalized least square regression models testing for the influence of organismal features on Edf-α in which raw estimates were substituted with 0 values when elevational trends were not fully significant. Rows report models, and column AICc, ΔAICc, and slopes of regressions with their SE and tests of significance. Models might include or not a phylogenetic structure based on the Brownian motion BM model of evolution. When "BM" appears in the list of predictors, it means that phylogeny is affecting the relationship between organismal features and diversity variables; when "BM" appears alone, the model includes the sole intercept and phylogenetic structure. Significant estimates are highlighted in bold. Only models within 5 AICc points from the best models are shown.

Model	AICc	ΔAICc	Predictors	Estimate	SE	t	P
Lichens: Edf-α (0 corr.)							
Endolithic + Algal symbiosis; BM	53.38	0.00	Endolithic	-6.061	7.65	0.79	0.445
			**Algal symbiosis**	**3.733**	**1.53**	**2.44**	**0.033**
Algal symbiosis; BM	55.86	2.48	**Algal symbiosis**	**3.404**	**1.45**	**2.35**	**0.037**
Endolithic + Algal symbiosis	56.98	3.60	Endolithic	-5.031	6.66	0.76	0.466
			**Algal symbiosis**	**3.732**	**1.33**	**2.82**	**0.017**
Endolithic; BM	57.18	3.80	Endolithic	-0.984	8.75	0.11	0.912
Algal symbiosis	59.13	5.75	**Algal symbiosis**	**3.391**	**1.22**	**2.77**	**0.017**
BM	60.02	6.64	-	-	-	-	-
Endolithic	61.80	8.43	Endolithic	1.377	7.87	0.18	0.864
Insects: Edf-α (0 corr.)							
Size-corrected wing length + Plant Interaction; BM	38.39	0.00	Size-corrected wing length	-6.099	2.65	2.30	0.061
			**Plant Interaction**	**3.460**	**1.30**	**2.65**	**0.038**
Size-corrected wing length; BM	38.91	0.52	Size-corrected wing length	-3.484	3.36	1.04	0.334
Plant interaction; BM	39.32	0.93	Plant Interaction	2.345	1.54	1.53	0.171
BM	39.45	1.06	-	-	-	-	-
Body size; BM	39.95	1.56	Body size	0.458	2.84	0.16	0.877

Size-corrected wing length + Plant Interaction	40.36	1.97	**Size-corrected wing length**	**-6.152**	**2.00**	**3.08**	**0.022**
			**Plant Interaction**	**4.153**	**1.14**	**3.63**	**0.011**
Plant Interaction	42.53	4.14	Plant Interaction	2.327	1.45	1.60	0.154
Birds: Edf-α (0 corr.)							
Plant interaction	37.34	0.00	Plant Interaction	4.352	2.30	1.89	0.101
Plant Interaction; BM	38.25	0.91	**Plant Interaction**	**5.230**	**1.55**	**3.38**	**0.012**
BM	39.28	1.94	-	-	-	-	-
Size-corrected wing length	39.10	1.76	Size-corrected wing length	-1.234	0.54	2.28	0.057
Size-corrected wing length; BM	40.40	3.06	**Size-corrected wing length**	**-1.713**	**0.50**	**3.42**	**0.011**
Body size; BM	41.31	3.97	Body size	-0.965	1.00	0.97	0.366

Supplementary References

1. Laiolo, P., Tella, J. L. Landscape bioacoustics allow detection of the effects of habitat patchiness on population structure. *Ecology* **87**, 1203-1214 (2006).

2. Quantum GIS Development Team. Quantum GIS Geographic Information (2012).

3. López de Silanes Vázquez, M. E., Alfonso, A. T., Etayo J. Líquenes y hongos liquenícolas de Fuentes Carrionas, Sierra de Riaño y Valle de Liébana (N de España). NACC: Nova acta científica compostelana. *Bioloxía* **8**, 47-89 (1997).

4. López de Silanes Vázquez, M. E., Paz-Bermudez G., Etayo J., Terrón A. Aportación al catálogo de líquenes del Parque Nacional de Los Picos de Europa, N de España. *Nova Acta Científica Compostelana (Bioloxia)* **9**, 83-98 (1999).

5. De La Torre Fernández, F., Fernández Ordóñez, M. C. Catálogo de líquenes en Asturias. *Acta Botánica Malacitana* **25**, 45-59 (2000).

6. Aragón, G., Martínez, I., Burgaz, A. R., Belinchón, R., Prieto, M., Otálora, M. A. et al.. Inventariación de la diversidad liquénica del Parque Nacional de los Picos de Europa. *Proyectos de Investigación en Parques Nacionales* **2006**, 77-104 (2003).

7. Laiolo, P., Seoane, J., Obeso, J. R. Illera, J. C. Ecological divergence among young lineages favours sympatry, but convergence among old ones allows coexistence in syntopy. *Global Ecology and Biogeography* **26**, 601-608 (2017).

8. Laiolo, P., Pato, J., Obeso, J. R. Ecological and evolutionary drivers of the elevational gradient of diversity. *Ecology letters* **21**, 1022-1032 (2018).

9. Ocharan Larrondo F. J. Los Odonatos de Asturias y España. Aspectos sistemáticos y faunísticos. PhD Dissertation. Oviedo University (1987).

10. González, M. A., Otero, J. C. Observaciones sobre los Tricópteros de la Península Ibérica. V. Tricópteros de los Picos de Europa (Norte de España). *Boletín de la Asociación Española de Entomología* **8**, 47-52 (1984).

11. González, M. A., Valiela, J., Cobo, F. Observaciones sobre los Tricópteros de la península ibérica. VIII: Sierras de Ancares y del Caurel (Noroeste de España)

(Trichoptera). *Boletín de la Asociación Española de Entomología* **13**, 331-341 (1989).

12. Braga, A. M. Utilizacion de macroinvertebrados benticos como indicadores biológicos de la calidad del agua en el rio Viao-Piloña (Asturias). *Limnética* **3**, 141-150 (1987).

13. Ocharan, R., Ocharan, F. J., Anadón, A. Tricópteros de la Reserva de la Biosfera de Muniellos y de Asturias (N de España). *Boletín de la Asociación Española de Entomología* **30**, 161-197 (2006).

14. Martínez, J., González, M. A. Tricópteros de los parques naturales de Somiedo y Las Ubiñas-La Mesa (Asturias, Norte de España) (Insecta:Trichoptera). *Boletín de la Asociación Española de Entomología* **35**, 231-248 (2011).

15. Martínez, J., Martín, L., González, M. A. Nuevos datos sobre los tricópteros (Insecta, Trichoptera) de Asturias (N. España). *Boletín de la Asociación Española de Entomología* **40,** 43-66 (2016).

16. Martín, L., Martínez, J., González, R. González, M. A. Tricópteros (Insecta, Trichoptera) de la Montaña Palentina (Parque Natural de las Fuentes Carrionas y Fuente Cobre) y de la sierra de La Cabrera (León). *Boletín de la Asociación Española de Entomología* **40,** 251-268 (2016).

17. Laiolo, P., Illera, J. C., Obeso, J. R. Local climate determines intra-and interspecific variation in sexual size dimorphism in mountain grasshopper communities. *Journal of Evolutionary Biology* **26**, 2171-2183 (2013).

18. Ornosa, C., Ortiz-Sánchez, F. J. Hymenoptera: Apoidea I (Vol. 23). *Editorial CSIC - CSIC Press* (2004).

19. Cayuela, L., Ruiz-Arriaga, S., Ozers, C. P. Honeybees increase fruit set in native plant species important for wildlife conservation. *Environmental management* **48**, 910-919 (2011).

20. Paradis, E. Analysis of Phylogenetics and Evolution with R, 2nd Edn. Springer, New York, NY (2012).

21. Kumar, S., Stecher, G., Suleski, M., Hedges, S. B. TimeTree: a resource for timelines, timetrees, and divergence times. *Mol. Biol. Evol.* **34**, 1812-1819 (2017).

22. Laiolo, P., Seoane, J., Illera, J. C., Bastianelli, G., Carrascal, L. M., Obeso J. R. The evolutionary convergence of avian lifestyles and their constrained coevolution with species' ecological niche. *Proc R Soc B* **282**, 20151808 (2015).

23. Slack, H. D. The food of caddis fly (Trichoptera) larvae. *Journal of Animal Ecology* **5**, 105-115 (1936).

24. Harding, D. J. L. Distribution and population dynamics of a litter-dwelling caddis, *Enoicyla pusilla* (Trichoptera). *Applied Soil Ecology* **9**, 203-208 (1998).

25. Presa, Y., Soto, J., Postigo, M. Estudio de los tricópteros del río Órbigo (León). *Scientia gerundensis* **16**, 203 (1990).

26. Lavandier, P., Cereghino, R. Use and partition of space and resources by two coexisting *Rhyacophila* species (Trichoptera) in a high mountain stream. In: Space Partition within Aquatic Ecosystems. Springer, Dordrecht, 157-162 (1995).

27. Cereghino, R. Shift from a herbivorous to a carnivorous diet during the larval development of some *Rhyacophila species* (Trichoptera). *Aquatic Insects* **24**, 129-135 (2002).

28. Basaguren, A., Riano, P., Pozo, J. Life history patterns and dietary changes of several caddisfly (Trichoptera) species in a northern Spain stream. *Archiv für Hydrobiologie* 23-41 (2002).

29. Corallini, C., Bicchierai, M. C. Trichoptera larvae and gregarines: Host-parasite relationships. *Zoosymposia* **10**, 148-164 (2016).

30. Galliani, C., Scherini, R., Piglia, A. Dragonflies and Damselflies of Europe: A scientific approach to the identification of European Odonata without capture (Vol. 7), WBA Project Srl. (2017).

31. Nimis P.L., Martellos S. ITALIC - The Information System on Italian Lichens. Version 5.0. University of Trieste, Dept. of Biology (http://dryades.units.it/italic). (2017).

32. Ahmadjian, V. The lichen photobiont: what can it tell us about lichen systematics?. *Bryologist* **96**, 310-313 (1993).

33. Rambold, G., Friedl, T., Beck, A. Photobionts in lichens: possible indicators of phylogenetic relationships?. *Bryologist* **101**, 392-397 (1998).

34. Beck, A., Friedl, T., Rambold, G.. Selectivity of photobiont choice in a defined lichen community: inferences from cultural and molecular studies. *New Phytologist* **139**, 709-720 (1998).

35. Helms, G., Friedl, T., Rambold, G., Mayrhofer, H.. Identification of photobionts from the lichen family Physciaceae using algal-specific ITS rDNA sequencing. *The Lichenologist* **33**, 73-86 (2001).

36. Meier, F. A., Scherrer, S., Honegger, R. Faecal pellets of lichenivorous mites contain viable cells of the lichen-forming ascomycete *Xanthoria parietina* and its green algal photobiont, *Trebouxia arboricola*. *Biological Journal of the Linnean Society* **76**, 259-268 (2002).

37. Dahlman, L., Persson, J., Näsholm, T., Palmqvist, K. Carbon and nitrogen distribution in the green algal lichens *Hypogymnia physodes* and *Platismatia glauca* in relation to nutrient supply. *Planta* **217**, 41-48 (2003).

38. Piercey-Normore, M. D. The lichen-forming ascomycete *Evernia mesomorpha* associates with multiple genotypes of *Trebouxia jamesii*. *New Phytologist* **169**, 331-344 (2006).

39. Thüs, H., Muggia, L., Pérez-Ortega, S., Favero-Longo, S. E., Joneson, S., O'Brien, H., et al. Revisiting photobiont diversity in the lichen family Verrucariaceae (Ascomycota). *European Journal of Phycology* **46**, 399-415 (2011).

40. Dal Grande, F., Beck, A., Cornejo, C., Singh, G., Cheenacharoen, S., Nelsen, M. P., Scheidegger, C. Molecular phylogeny and symbiotic selectivity of the green algal genus *Dictyochloropsis* sl (Trebouxiophyceae): a polyphyletic and widespread group forming photobiont-mediated guilds in the lichen family Lobariaceae. *New Phytologist* **202**, 455-470 (2014).

41. Lindgren, H., Velmala, S., Högnabba, F., Goward, T., Holien, H., Myllys, L. High fungal selectivity for algal symbionts in the genus *Bryoria*. *The Lichenologist* **46**, 681-695 (2014).

42. Leavitt, S. D., Kraichak, E., Nelsen, M. P., Altermann, S., Divakar, P. K., Alors, D., et al. Fungal specificity and selectivity for algae play a major role in determining lichen partnerships across diverse ecogeographic regions in the lichen-forming family Parmeliaceae (Ascomycota). *Molecular Ecology* **24**, 3779-3797 (2015).

43. Sadowsky, A., Ott, S. Symbiosis as a successful strategy in continental Antarctica: performance and protection of *Trebouxia* photosystem II in relation to lichen pigmentation. *Polar Biology* **39**, 139-151 (2016).

44. Voytsekhovich, A., Beck, A. Lichen photobionts of the rocky outcrops of Karadag massif (Crimean Peninsula). *Symbiosis* **68**, 9-24 (2016).

45. Moya, P., Chiva, S., Molins, A., Jadrná, I., Škaloud, P., Peksa, O., Barreno, E. *Myrmecia israeliensis* as the primary symbiotic microalga in squamulose lichens growing in European and Canary Island terricolous communities. *Fottea* **18**, 72-85 (2018).
