# Supplementary material for: Evolutionary conservation of within-family biodiversity patterns
Source: Nat Commun. 2020 Feb 14;11:882. doi: 10.1038/s41467-020-14720-3 (PMC7021778; doi:10.1038/s41467-020-14720-3)
Supplement: Supplementary file 3 — Reporting Summary [file 41467_2020_14720_MOESM3_ESM.pdf]

nature research

Corresponding author(s): NCOMMS-19-16515

Last updated by author(s): YYYY-MM-DD

Reporting Summary

Nature Research wishes to improve the reproducibility of the work that we publish. This form provides structure for consistency and transparency in reporting. For further information on Nature Research policies, see Authors & Referees and the Editorial Policy Checklist .

Statistics

For all statistical analyses, confirm that the following items are present in the figure legend, table legend, main text, or Methods section.

n/a	Confirmed	
☐	☒	The exact sample size (*n*) for each experimental group/condition, given as a discrete number and unit of measurement
☐	☒	A statement on whether measurements were taken from distinct samples or whether the same sample was measured repeatedly
☐	☒	The statistical test(s) used AND whether they are one- or two-sided<br>*Only common tests should be described solely by name; describe more complex techniques in the Methods section.*
☐	☒	A description of all covariates tested
☐	☒	A description of any assumptions or corrections, such as tests of normality and adjustment for multiple comparisons
☐	☒	A full description of the statistical parameters including central tendency (e.g. means) or other basic estimates (e.g. regression coefficient) AND variation (e.g. standard deviation) or associated estimates of uncertainty (e.g. confidence intervals)
☐	☒	For null hypothesis testing, the test statistic (e.g. *F*, *t*, *r*) with confidence intervals, effect sizes, degrees of freedom and *P* value noted<br>*Give P values as exact values whenever suitable.*
☒	☐	For Bayesian analysis, information on the choice of priors and Markov chain Monte Carlo settings
☒	☐	For hierarchical and complex designs, identification of the appropriate level for tests and full reporting of outcomes
☐	☒	Estimates of effect sizes (e.g. Cohen's *d*, Pearson's *r*), indicating how they were calculated

Our web collection on statistics for biologists contains articles on many of the points above.

Software and code

Policy information about availability of computer code

Data collection	-
Data analysis	R Core Team (2018) R: A Language and Environment for Statistical Computing. R Foundation for Statistical Computing, Vienna. https://www.R-project.org

For manuscripts utilizing custom algorithms or software that are central to the research but not yet described in published literature, software must be made available to editors/reviewers. We strongly encourage code deposition in a community repository (e.g. GitHub). See the Nature Research guidelines for submitting code & software for further information.

Data

Policy information about availability of data

All manuscripts must include a data availability statement. This statement should provide the following information, where applicable:

- Accession codes, unique identifiers, or web links for publicly available datasets
- A list of figures that have associated raw data
- A description of any restrictions on data availability

Source data underlying this study are provided as supplementary material (data points for Figure 2 and 3). Data are available in the Digital CSIC repository, http://dx.doi.org/10.20350/digitalCSIC/10529

Field-specific reporting

Please select the one below that is the best fit for your research. If you are not sure, read the appropriate sections before making your selection.

Ecological, evolutionary & environmental sciences study design

All studies must disclose on these points even when the disclosure is negative.

Study description	Analysis of Phylogenetic patterns in community variables and organismal and higher level traits [Monte Carlo simulations , Blomberg K, and regressions on distance matrices] and Relationships between community variables and organismal and higher level traits [generalized least square regressions]. Sample size is 9 (insects, birds) and 14 (lichens) for these analyses.<br><br>Community variables were estimated with generalized additive models, piecewise regressions and multiple regressions on distance matrices. Sample size (number of occupied plots) varies from 50 to over 1546 (Detail in Fig. 2).
Research sample	We use presence absence of bird, lichen and insect species in the Cantabran Mountains region (N Spain). Data were collected by ourselves (insects, birds) and obtained from literature (insects, lichens).<br>Information on traits was obtained from own measurements (insects) and literature (insects, birds, lichens).<br>Phylogenetic information was obtained from literature.
Sampling strategy	Surveys: Area counts (birds), captures with nets (grasshoppers, bees)<br>Morphometric mesurements were taken with a binocular microscope
Data collection	Part of the data were obtained from literature. For own data: bird surveys were performed by Paola Laiolo & José Ramón Obeso; grasshopper surveys were performed by Paola Laiolo, José Ramón Obeso and Joaquina Pato; bee surveys were perfomed by Paola Laiolo & Joaquina Pato. Grasshoppers and bees were identified by Paola Laiolo & Joaquina Pato.
Timing and spatial scale	Our own data were collected starting from 2008 (up 2018). The study was performed in the Cantabrian Mountain range up to the Atlantic coast of Asturias.
Data exclusions	No data were excluded
Reproducibility	For our own data, insect collections are available in our Research Institute (Research Unit of Biodiversity, Campus de Mieres, Spain)
Randomization	N.A.
Blinding	Community data were collected for purposes distinct from this study

Did the study involve field work? ☒ Yes ☐ No

Field work, collection and transport

Field conditions	Birds were surveyed in spring, tracking bird breeding phenology along the elevation gradient, beginning the fieldwork at the end of March in lowlands and ending in July in the highlands, and monitoring birds form sunrise to midday in good weather conditions only. Insects were surveyed in late May-September, from mid – late morning up to mid-afternoon, in favourable (dry, sunny, no windy) weather conditions
Location	The study was performed in a region comprised between Latitude 42.76º and 43.65º, and Longitude -4.40º and -7.05º in NW Spain. The altitudinal range of the Cantabrian Mountains spans from 0 to 2648 m a.s.l..
Access and import/export	Bird observations were performed with non invasive standard methods (visual and aural detection).<br>Insects were collected with the following permissions: CO/09/169/2018, CO/09/242/2017, CO/09/0140/2016 (Gobierno del Principado de Asturias - Picos de Europa National Park); 2016/025703, 2017/021921, 2017/020246 (Gobierno del Principado de Asturias - Regional Natural Parks).
Disturbance	In the case of insects, sampling was performed during periods of peak abundance, our collection involved a very small portion of the individuals available in each sampling plots. In the case of birds, we limited our activity to observing/listening.

Reporting for specific materials, systems and methods

We require information from authors about some types of materials, experimental systems and methods used in many studies. Here, indicate whether each material, system or method listed is relevant to your study. If you are not sure if a list item applies to your research, read the appropriate section before selecting a response.

Materials & experimental systems

n/a	Involved in the study
☒	☐ Antibodies
☒	☐ Eukaryotic cell lines
☒	☐ Palaeontology
☐	☒ Animals and other organisms
☒	☐ Human research participants
☒	☐ Clinical data

Methods

n/a	Involved in the study
☒	☐ ChIP-seq
☒	☐ Flow cytometry
☒	☐ MRI-based neuroimaging

Animals and other organisms

Policy information about studies involving animals; ARRIVE guidelines recommended for reporting animal research

Laboratory animals	No laboratory animal was used
Wild animals	Birds were observed. Insects were collected with regular permissions. No species of conservation concern was collected.
Field-collected samples	Field-collected insects were frozen upon return to the laboratory, and form part of the collections of the Research Unit of Biodiversity.
Ethics oversight	No ethical approval was necessary for our work.

Note that full information on the approval of the study protocol must also be provided in the manuscript.
